# Supplementary material for: Different In Vitro-Generated MUTZ-3-Derived Dendritic Cell Types Secrete Dexosomes with Distinct Phenotypes and Antigen Presentation Potencies
Source: Int J Mol Sci. 2022 Jul 28;23(15):8362. doi: 10.3390/ijms23158362 (PMC9368791; doi:10.3390/ijms23158362)
Supplement: Supplementary file 1 [file ijms-23-08362-s001.zip › ijms-1780187-supplementary.pdf]

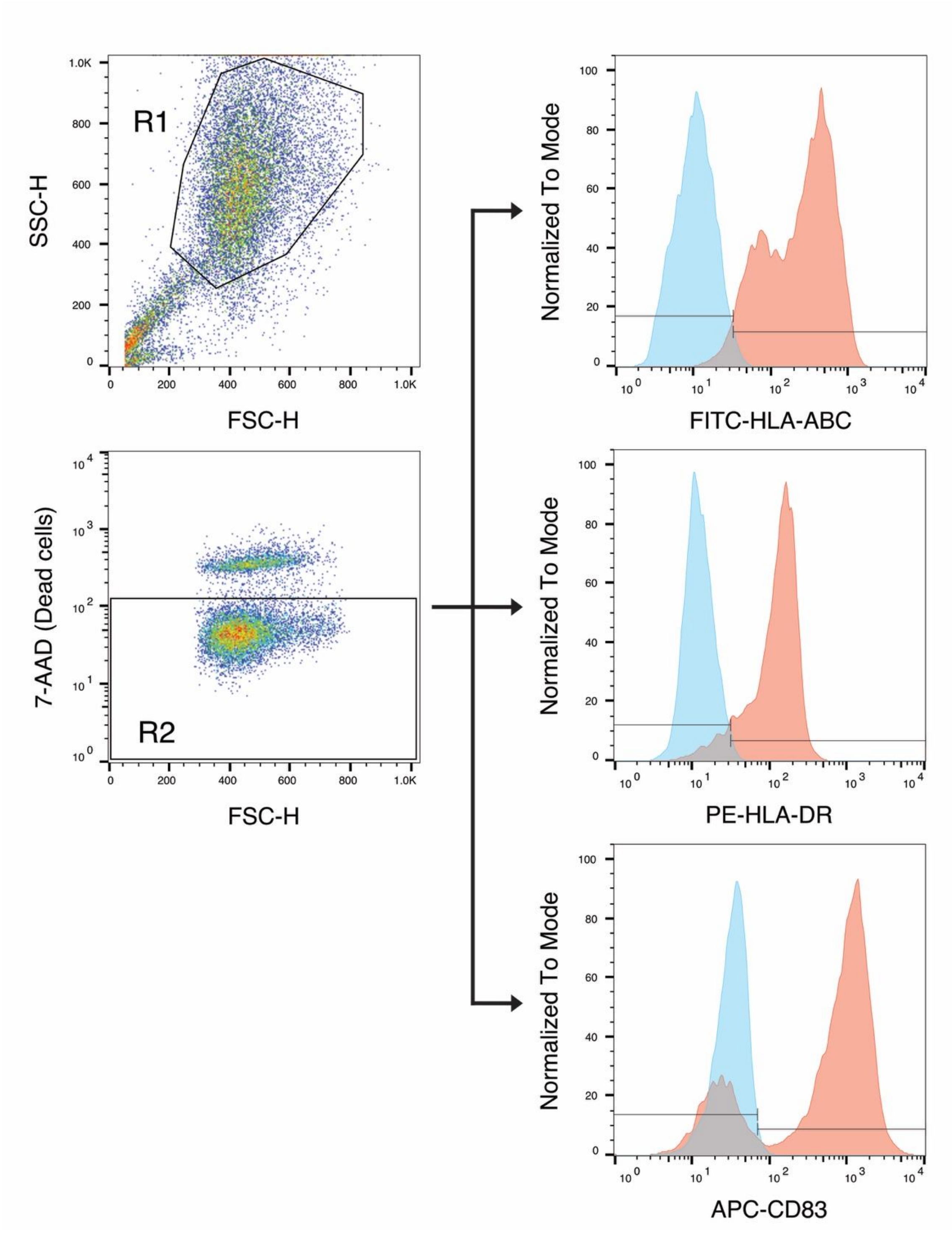

Figure S1. Gate strategy for the identification of MUTZ-3 derived IL-4- and IFN-DCs. The frequency of positive cells was analyzed using the Flowjo software in two regions after staining the cells with each antibody for DC maturation markers, MUTZ-3-derived DCs (R1) gated on forward scatter (FSC) and side scatter (SSC). Live

cells gated on FSC and 7−AAD without dead cells were examined for immunophenotyping (R2). The right panel indicates the percentage of HLA-ABC (FITC), HLA-DR (PE), and CD83 (APC) histogram analyses. The percentages in the panels indicate the positive ratio of each marker in live DCs. The red and blue histograms indicate the cells stained with each antibody (FITC, PE, and APC) and the isotype control, respectively. The histograms have been normalized to the modal values.

Table S1. HLA types of the used MUTZ-3 cells. The table presents the HLA types of the MUTZ-3 cells used in this study.

		MUTZ-3 HLA typing	
HLA type		Allele 1	Allele 2
HLA-class I	A	**02:01**	03:01
	B	44:02	44:03
	C	07:04	04:01
HLA-class II	DRB1	10:01	11:01
	DQA1	01:05	05:05
	DQB1	05:01	03:01
	DPB1	01:03	-
	DPB2	03:01	04:01

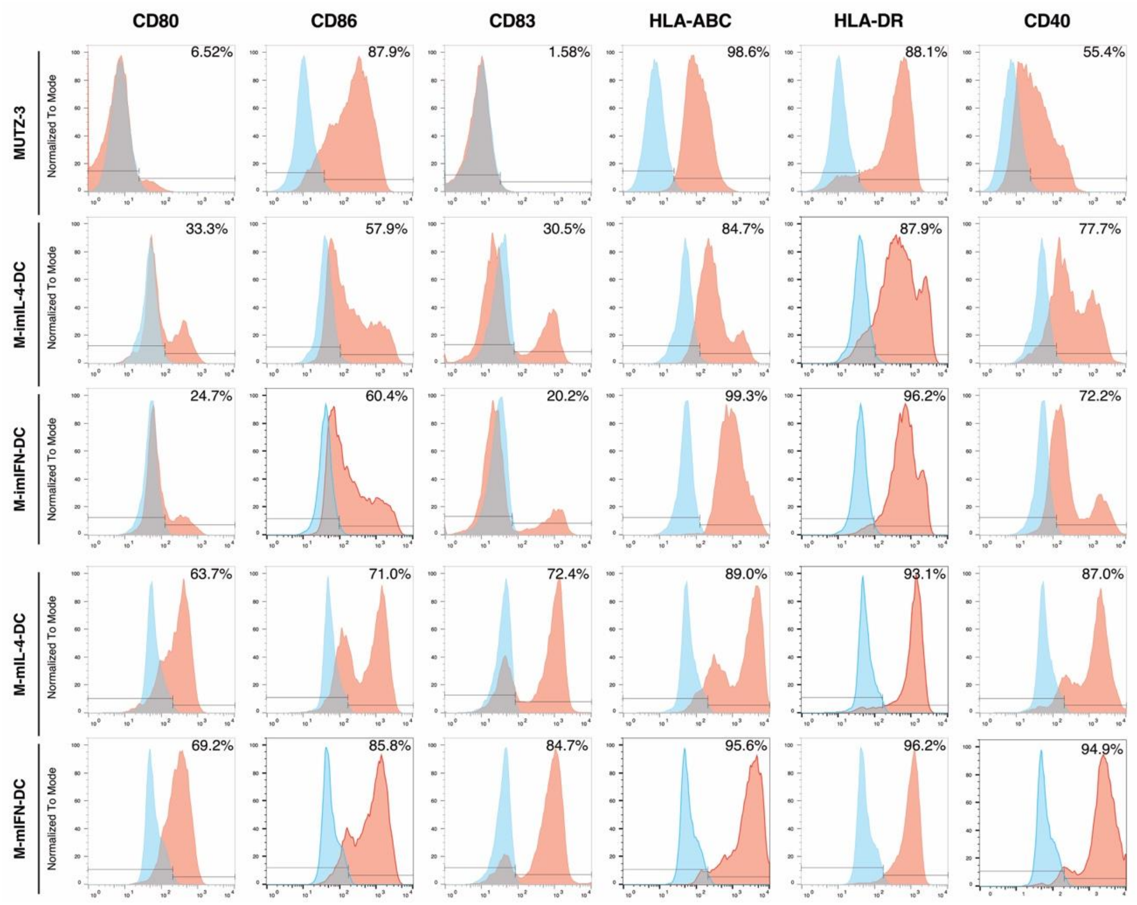

Figure S2. DC maturation-associated cell surface marker expression in MUTZ-3, immature, and mature MUTZ-3-derived IL-4- and IFN-DCs. Representative FACS histograms with isotype control for the presented markers in MUTZ-3, M-imIL-4-DC, M-imIFN-DC, M-mIL-4-DC, and M-mIFN-DC (CD80, CD86, CD83, HLA-ABC, HLA-DR, and CD40). The percentages in the panels indicate the positive ratio of each marker. The red and blue histograms indicate cells stained with each antibody and isotype-control, respectively. The histograms have been normalized to the modal values.

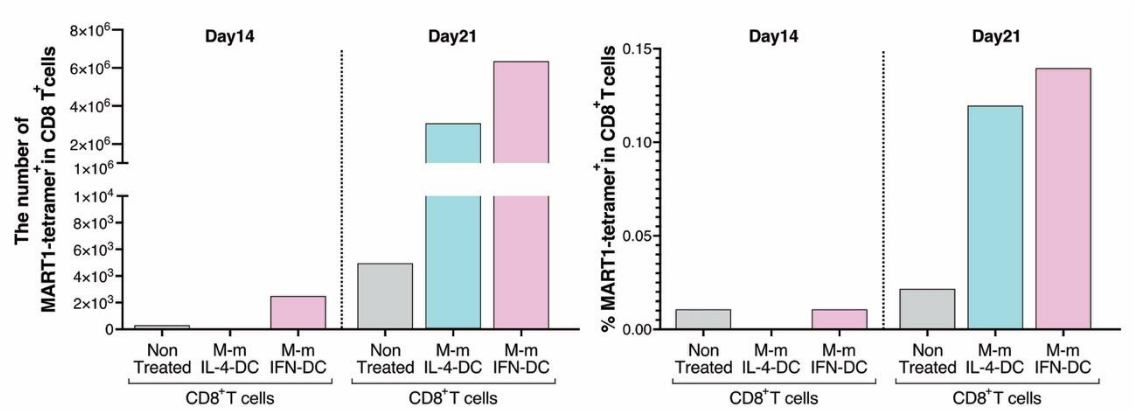

Figure S3. Comparison of MART-1-specific induction of antigen presentation in mature MUTZ-3-derived IL-4- and IFN-DCs. M-mIL-4-DCs or M-mIFN-DCs were co-cultured with autologous T cells at a DC:_T cell ratio of 1:10. MART-1-specific CD8$^+$ T cells were detected using CD3$^+$, CD8$^+$, and MART-1$^+$ tetramers via flow cytometry 14 and 21 days after the start of co-culture. The bar graphs present the number of MART-1 tetramer$^+$/CD8$^+$ T cells and the MART-1 tetramer$^+$ ratio in CD8$^+$ T cells during the culture period.

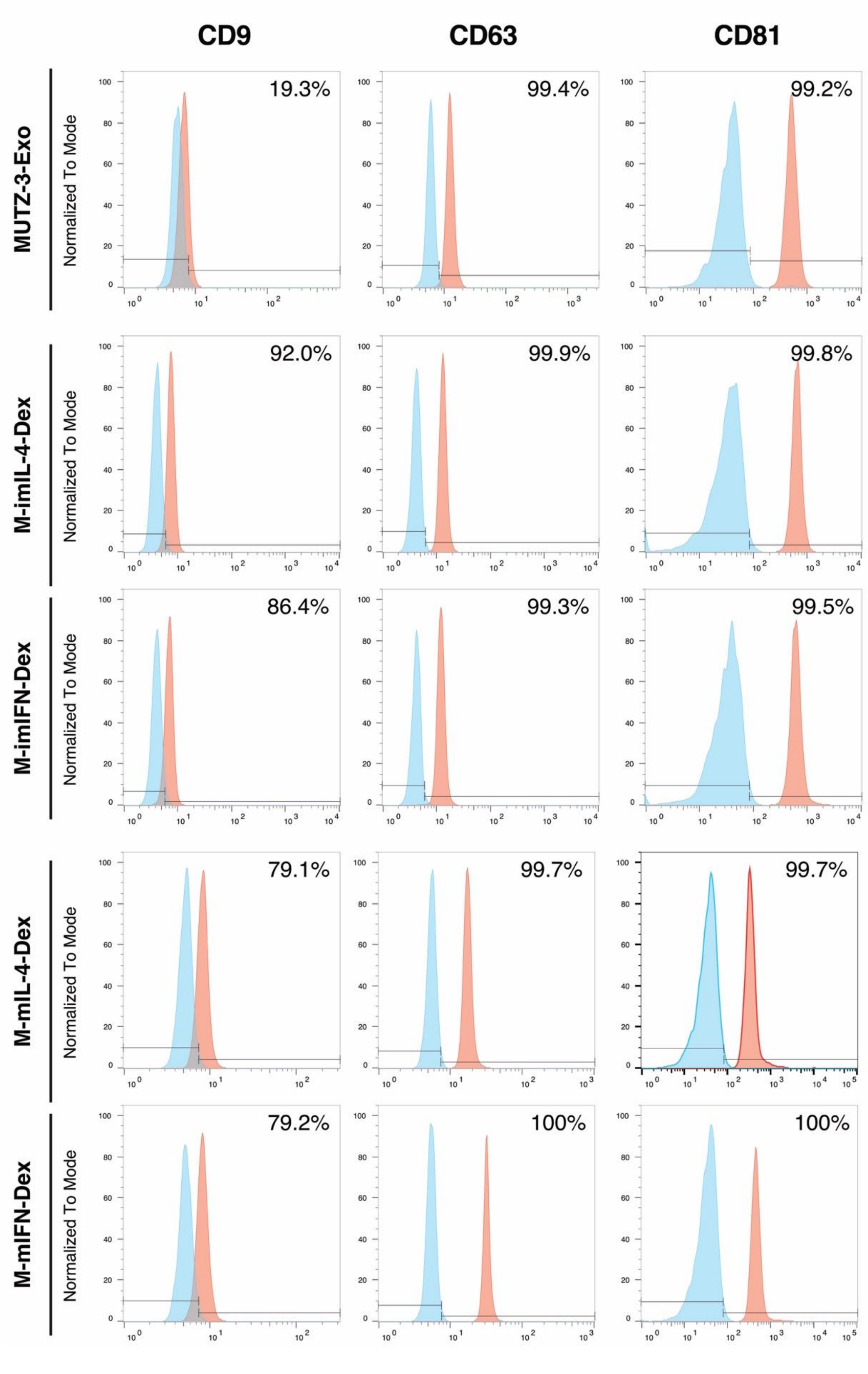
CD9
CD63
CD81
MUTZ-3-Exo
M-imIL-4-Dex
M-imIFN-Dex
M-mIL-4-Dex
M-mIFN-Dex
Normalized To Mode
19.3%
99.4%
99.2%
92.0%
99.9%
99.8%
86.4%
99.3%
99.5%
79.1%
99.7%
99.7%
79.2%
100%
100%

(a)

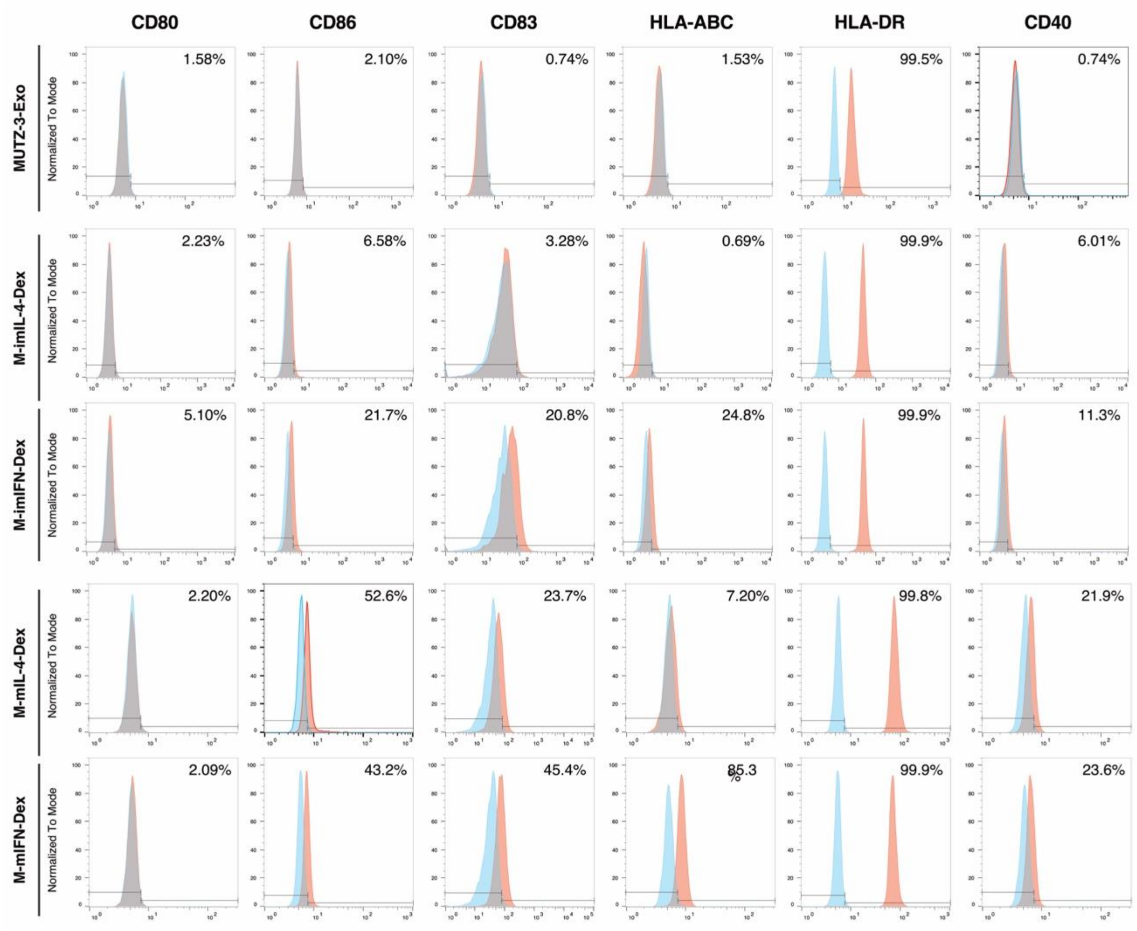

(**b**)

Figure S4. The expressions of exosome- and DC maturation-associated cell surface markers in MUTZ-3-Exosome and MUTZ-3-derived IL-4- and IFN-DC-Dexosome. (**a**) Representative FACS histograms of the exosome markers (CD9, CD63, and CD81) in MUTZ-3-Exosome (MUTZ-3-Exo), immature, and mature MUTZ-3-derived IL-4 or IFN-DC-Dexosomes (M-imIL-4-Dex, M-imIFN-Dex, M-mIL-4-Dex, and M-mIFN-Dex). (**b**) Representative FACS histograms with isotype control for the presented markers (CD80, CD86, CD83, HLA-ABC, HLA-DR, and CD40). The percentages in the panels indicate the positive ratio of each marker. The red and blue histograms indicate the MUTZ-3-Exo, M-imIL-4-Dex, M-imIFN-Dex, M-mIL-4-Dex, and M-mIFN-Dex stained by each antibody and the isotype-control, respectively. The histograms have been normalized to the modal values.

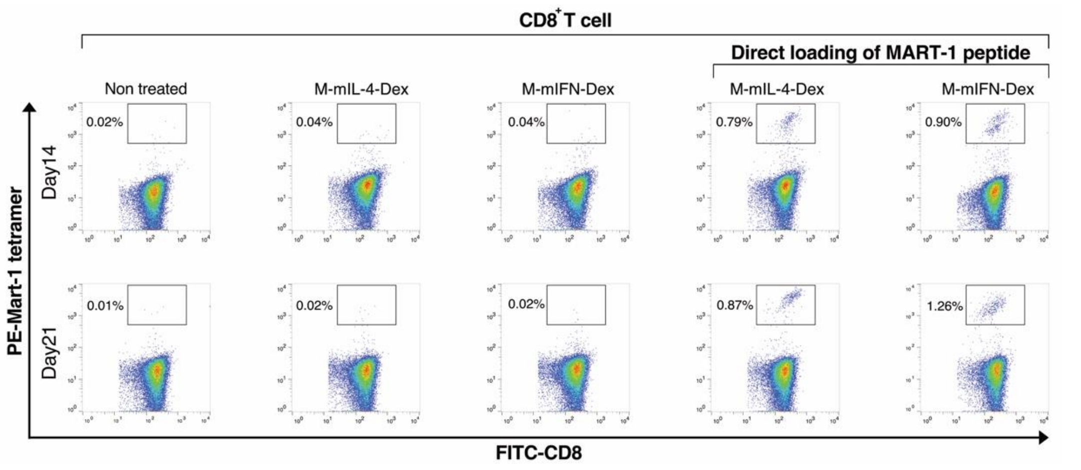
CD8+ T cell
Direct loading of MART-1 peptide
Non treated
M-mIL-4-Dex
M-mIFN-Dex
M-mIL-4-Dex
M-mIFN-Dex
Day14
Day21
0.02%
0.04%
0.04%
0.79%
0.90%
0.01%
0.02%
0.02%
0.87%
1.26%
PE-Mart-1 tetramer
FITC-CD8

(a)

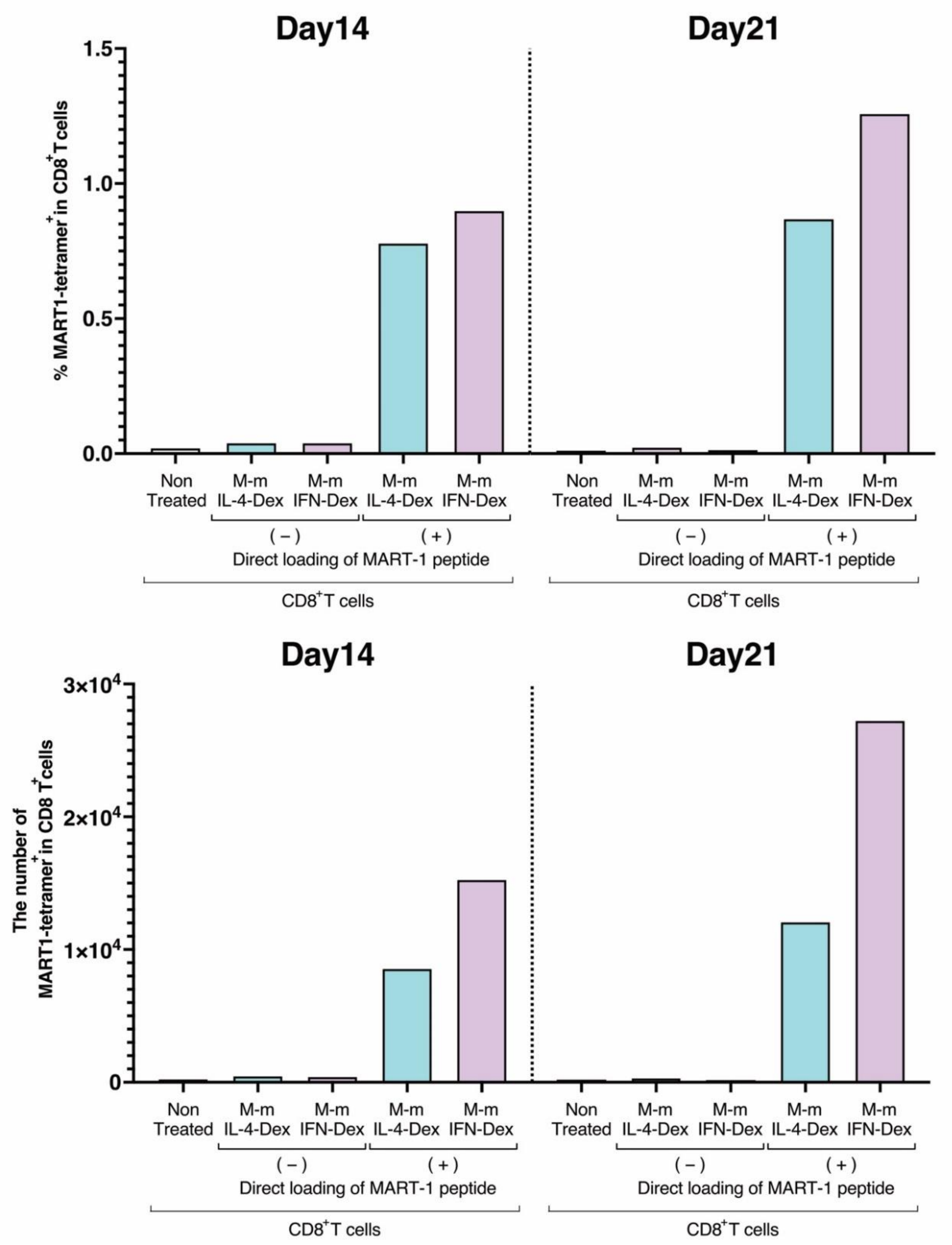

(b)

Figure S5. Comparison of MART-1-specific induction of antigen presentation ability in direct MART-1 peptide loading on mature MUTZ-3-derived IL-4- and IFN-DC-dexosomes. M-mIL-4-Dex or M-mIFN-Dex was co-cultured with autologous T cells at a Dexosome: T cell ratio of 2000: 1. (a) Dot plots of a

representative example. The percentages in the panels indicate the MART-1 tetramer$^+$ ratio in CD8$^+$ T cells. (b) The bar graphs present the number of MART-1 tetramer$^+$/CD8$^+$ cells and the MART-1 tetramer$^+$ ratio in CD8$^+$ T cells during the culture period.
